# Passive acoustic monitoring for detecting the Yellow-bellied Glider, a highly vocal arboreal marsupial

**DOI:** 10.1371/journal.pone.0252092

**Published:** 2021-05-25

**Authors:** Desley A. Whisson, Freya McKinnon, Matthew Lefoe, Anthony R. Rendall

**Affiliations:** Centre for Integrative Ecology, School of Life and Environmental Sciences, Deakin University, Burwood, Victoria, Australia; Wildlife Conservation Society Canada, CANADA

## Abstract

Passive acoustic monitoring (PAM) is increasingly being used for the survey of vocalising wildlife species that are otherwise cryptic and difficult to survey. Our study aimed to develop PAM guidelines for detecting the Yellow-bellied Glider, a highly vocal arboreal marsupial that occurs in native *Eucalyptus* forests in eastern and south-eastern Australia. To achieve this, we considered the influence of background noise, weather conditions, lunar illumination, time since sunset and season on the probability of detecting vocalisations. We deployed Autonomous Recording Units (ARUs) at 43 sites in the Central Highlands of Victoria during two periods: spring/summer (October 2018 to January 2019), and autumn/winter (May to August 2019). ARUs were programmed to record for 11 hours from sunset for 14 consecutive days during each period. Background noise resulted from inclement weather (wind and rain) and masked vocalisations in spectrograms of the recordings, thus having the greatest influence on detection probability. Vocalisations were most common in the four hours after sunset. Rainfall negatively influenced detection probability, especially during the autumn/winter sampling period. Detection of Yellow-bellied Gliders with PAM requires deploying ARUs programmed to record for four hours after sunset, for a minimum of six nights with minimal inclement weather (light or no wind or rain). The survey period should be extended to 12 nights when rain or wind are forecast. Because PAM is less labour intensive than active surveys (i.e., spotlighting and call playbacks with multiple observers and several nights’ survey per site), its use will facilitate broad-scale surveys for Yellow-bellied Gliders.

## Introduction

The imperfect detection of wildlife remains a challenge for surveying most species, particularly those species that are cryptic, have large home ranges, and occur at low densities or in inaccessible areas [1]. Recording a ‘false absence’ is a key issue associated with many survey methods, occurring by random chance, or due to survey design, observer experience or weather conditions [2, 3]. False absences can have significant consequences for species-habitat analyses resulting in ineffective impact assessments and poor implementation of conservation measures [4, 5]. The selection of a survey method is therefore a critical decision that not only influences the accuracy of the data collected, but also research and management outcomes [6].

In recent years, remote sensing methods such as camera trapping (see [7] for a review), and thermal infrared surveys with Unmanned Aerial Vehicles (e.g. [8]) have become more popular for wildlife survey. Passive acoustic monitoring (PAM) is a remote sensing method that is being applied more frequently for the survey of species that conspicuously advertise their presence, identity, and behavioural traits through vocalisations [9, 10]. With this method, autonomous recording units (ARUs) programmed to record when the species of interest is likely to be vocalising, are deployed at a site for an extended survey period [11, 12]. Recordings are then searched (either manually or with an automated detection and classification algorithm) for vocalisations of the species. PAM is effective for use in difficult terrain and vegetation, can be applied across large spatial and temporal scales, is non-invasive, and may improve detection probability of species that are small, nocturnal, elusive or uncommon [1, 12–14].

PAM has long been used for a wide range of taxa including birds [13, 14], bats [15], amphibians [16, 17], whales [18] and dolphins [19]. However, its application to determining presence or abundance of vocalising terrestrial mammals is relatively recent. Terrestrial mammals for which PAM has been applied include chimpanzees (*Pan troglodytes*; [20]), Golden Jackals (*Canis aureus*; [21]), Sika Deer (*Cervus nippon*; [22]) and koalas [23, 24]. There is considerable potential to develop PAM for other terrestrial mammals for which traditional survey methods are logistically challenging or result in high rates of false absences. In Australia, PAM may be effective for detecting highly vocal arboreal possum and glider species including the Yellow-bellied Glider (*Petaurus australis*), a species that is listed as ‘Near Threatened’ by the IUCN [25], and that lives in areas subject to increasing anthropogenic activity.

The Yellow-bellied Glider is a medium-sized (400–750 g) arboreal, gliding marsupial that has a widespread but patchy distribution in eastern and south-eastern mainland Australia [26]. It inhabits mature *Eucalyptus* forests and woodlands that provide tree hollows for denning, and food resources (eucalypt sap, nectar, pollen, manna, honeydew, and invertebrates). Yellow-bellied Gliders live in small family groups of two to six individuals which have large ranges of between 25 and 85 hectares encompassing dispersed and seasonally varying food resources, and with little overlap between family groups [27–30]. Consequently, they may occur at low population densities (0.06/hectare) even in suitable habitat [29–31].

The Yellow-bellied Glider is one of Australia’s most vocal marsupials, with vocalisations thought to play a role in territory defence [32]. Gliders call as they travel in pairs or groups between food trees, resulting in a positive correlation between gliding activity and calling rate [32, 33]. A typical full call lasts for up to four seconds and comprises two shrieks and a long gurgle. These calls are loud, distinctive, and can be heard by human observers at distances of up to 400 m [33–35]. Shorter calls of 0.75 seconds and comprising moans and gurgles also occur [33]. Calling activity is usually most frequent during the first three hours after dusk, corresponding to the period when gliders are emerging from their dens [32, 33, 36]. It may vary between nights due to rainfall [34], temperature [4], moon brightness [4], and the type of food resources being utilised [34]. On nights of heavy rain, Yellow-bellied Gliders often remain in their dens and therefore do not call [34]. When feeding on highly clumped food resources (i.e., sap, honeydew, manna, or nectar), Yellow-bellied Gliders spend little time gliding, thus resulting in less calling [34].

‘Active’ but not passive listening for Yellow-bellied Glider vocalisations or calls has long been used as a method for determining Yellow-bellied Glider presence or abundance at a site [4, 32, 33, 37, 38]. Two nocturnal survey methods are commonly used: (1) broadcasting Yellow-bellied Glider calls or Powerful Owl calls and listening for a response, and (2) listening for calls while searching with spotlights for Yellow-bellied Gliders (either a timed search or searching in a fixed area or along a transect). Multiple surveys per site have been recommended due to the potential influence of weather conditions and moonlight on Yellow-bellied Glider calling activity [37]. Consequently, these survey methods have high labour costs, and nocturnal surveys carry risks for observers. Developing a cost-effective survey method to determine presence of Yellow-bellied Gliders at a site is important for understanding the impacts of anthropogenic disturbance on the species, and for identifying population declines [38].

The aim of our study was to develop guidelines for using PAM for presence/absence surveys of Yellow-bellied Gliders, based on an understanding of the factors influencing Yellow-bellied Glider calling behaviour, and factors influencing the probability of detecting calls in recordings. Based on previous Yellow-bellied Glider research, we considered that detection probability would be influenced by time of night, weather conditions (wind, rain, and temperature), moonlight, and season (due to its influence on food supply and Yellow-bellied Glider behaviour). We also considered the effect of ambient noise due to inclement weather, on our ability to detect vocalisations in recordings.

## Materials and methods

### Study area

We conducted our study in the Toolangi State Forest in the Central Highlands of Victoria (37.5377° S 145.5189° E). The area comprises tall wet or damp *Eucalyptus* forest that is heavily logged (clearfell and salvage logging methods), resulting in a mosaic of different forest ages [39, 40]. The mean annual rainfall for the area is 1352.1 mm, with mean minimum and maximum temperatures of 7.4 and 15.8° C respectively [41].

### Bioacoustic surveys

We selected 43 sites with a minimum of 800 m between them to ensure that a Yellow-bellied Glider calling at one site would not be detected at a neighbouring site. This distance was chosen based on reports of Yellow-bellied Glider calls carrying up to 400 m [32]. All sites were surveyed from October 2018 to January 2019 (spring/summer) and from May to August 2019 (autumn/winter).

At each site, one ARU (Songmeter: SM4, Wildlife Acoustics, Maynard, Massachusetts, USA) was strapped to a tree, at 1.5 m above the ground, and left in place for one sampling period of 14 consecutive days in each season. This deployment period was chosen as feasible for a standard survey method and long enough to detect Yellow-bellied Gliders if present. ARUs comprised two built-in omnidirectional, low-noise stereo microphones, were powered with four internal batteries (1.5 V, D-Cell, alkaline), and fitted with Secure Digital (SD) cards (two 32 GB cards or one 64 GB card). Sample rate was set to 24 kHz, gain to 16 dB, and no high-pass filters were specified. ARUs were programmed to record continuously from 19:00 h to 07:00 h in spring/summer 2018, and from 17:00 h to 04:00 h in autumn/winter 2019. We reduced the period of recording per night during autumn/winter by one hour to ensure that we had enough battery power for a 14-day sampling period. The literature on Yellow-bellied Glider calling activity and our preliminary analysis of the spring/summer recordings suggested that we would most likely detect vocalisations in the earlier hours of the evening. Recordings were saved as one-hour files.

For each night of ARU deployment, we recorded lunar illumination (%), minimum and maximum nightly temperature (°C), and rainfall (mm). These variables are known to influence the activity of Yellow-bellied Gliders [4, 32, 33, 36, 42]. Lunar illumination was calculated through the ’lunar’ package in R [43] and represents the proportion of lunar illumination for any specific date and time. We calculated the lunar illumination at midnight of each sampling occasion. Data for minimum and maximum nightly temperatures and rainfall in 30-min intervals for the three closest weather stations (Scoresby Research Institute, Ferny Creek and Coldstream) were purchased from the Bureau of Meteorology. We averaged nightly data for each variable across all three stations and determined mean minimum and maximum temperatures and maximum rainfall for each sampling night.

### Audio data processing

**Fig 1:**
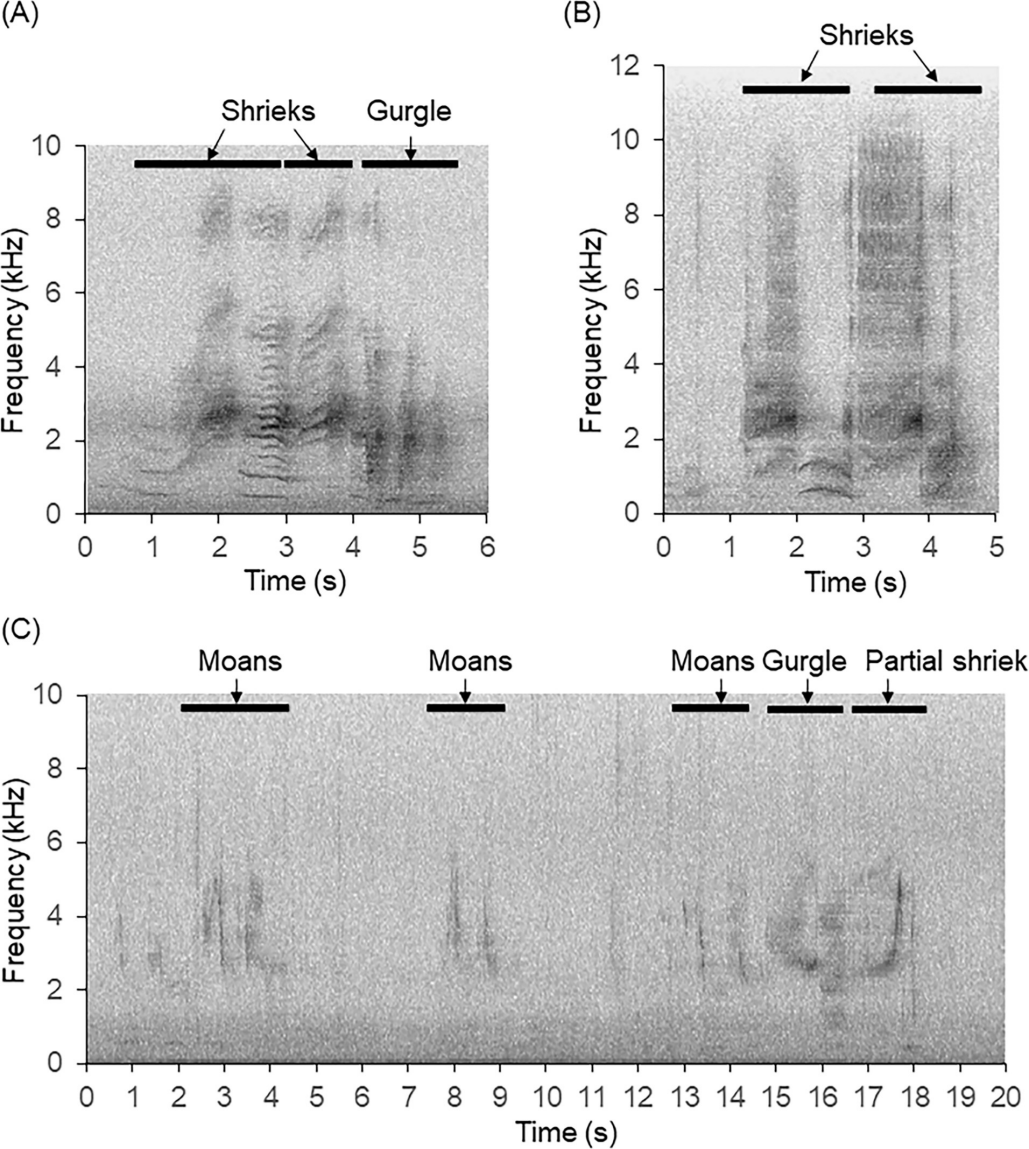
Spectrograms showing the variation in Yellow-bellied Glider vocalisations, including (A) full vocalisations comprising two shrieks and a long gurgle, given when not gliding, (B) non-gliding shrieks only, and (C) several moans, usually given just after gliding out of a tree, followed by a partial shriek [33].

**Fig 2:**
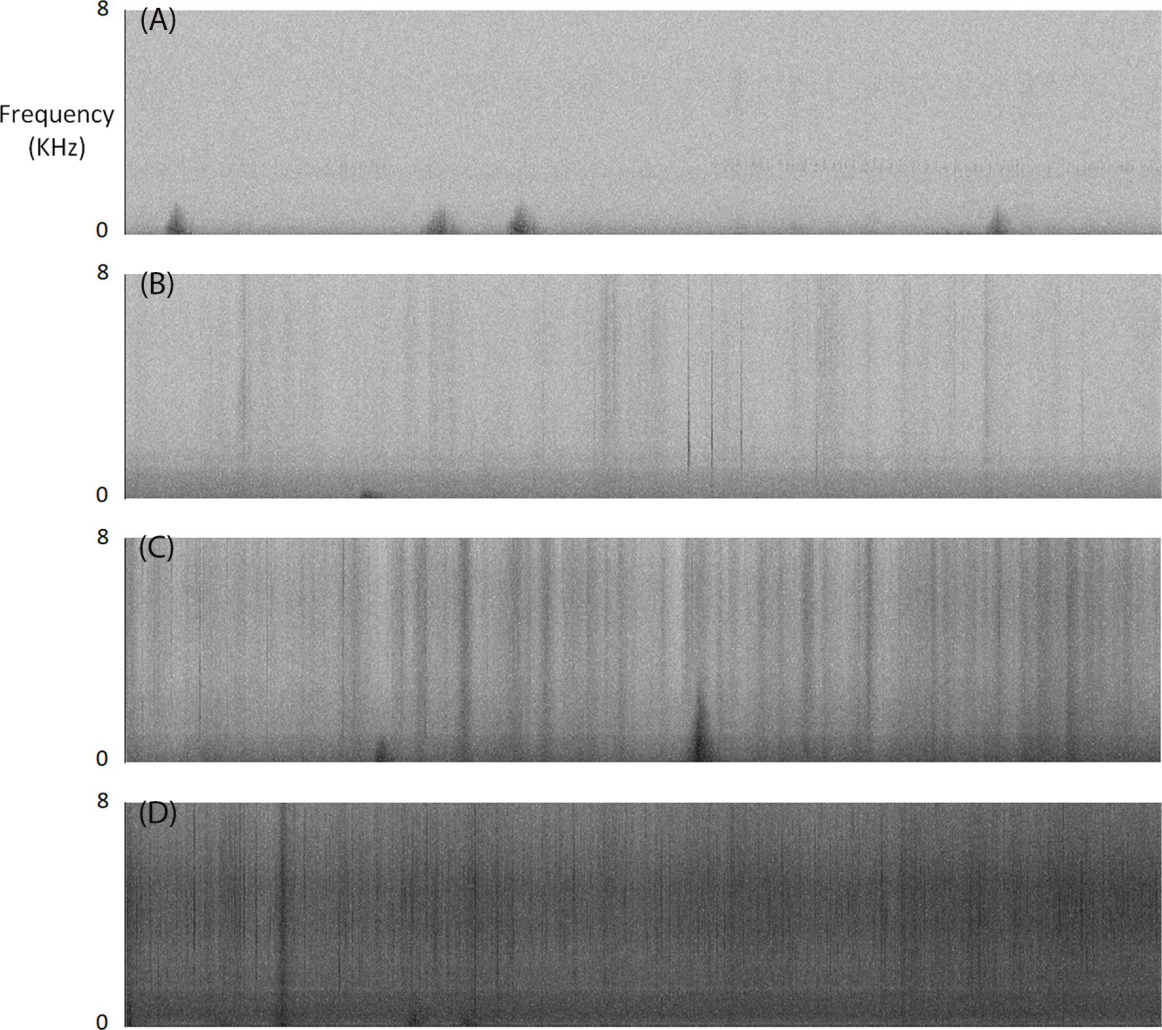
Spectrograms of recordings (one-hour duration) showing the influence of weather conditions on spectrogram clarity: (A) calm conditions with 0% spectrogram containing ambient noise, inclement weather score (IWS) = 0; (B) wind and rain causing ambient noise in 1–20% of spectrogram, IWS = 1; (C) wind and rain causing high levels of ambient noise in 21–50% of spectrogram, IWS = 2; and (D) wind and rain causing high levels of ambient noise in >50% of spectrogram, IWS = 3.

We manually searched spectrograms of our recordings to find Yellow-bellied Glider vocalisations (Fig 1; Audacity, Version 2.1.1, http://audacity.sourceforge.net/). We used a Hanning window with a 1024 Hz window size. We used the ‘Frequency’ algorithm with a linear scale of 0 to 8000 Hz, gain set to 20 dB and frequency gain set to 0 dB/dec. We viewed spectrograms in a 5-minute window and listened to any noise that might be Yellow-bellied Glider vocalisations. We chose these settings based on previous research with koalas [23] and after viewing a sample of recordings that contained Yellow-bellied Glider vocalisations. We recorded presence/absence of vocalisations per hour. We scored inclement weather for each hour from 0 to 3 (0 = calm conditions with 0% spectrogram containing ambient noise, 1 = wind and rain causing ambient noise in 1–20% of spectrogram, 2 = wind and rain causing high levels of ambient noise in 21–50% of spectrogram; and 3 = wind and rain causing high levels of ambient noise in >50% of spectrogram; Fig 2). We averaged the hourly scores for each night to provide a nightly inclement weather score.

### Data analysis

We used a Generalised Additive Mixed Model (GAMM) with a binomial distribution to examine the influence of time of night, season, and the interaction of time and season on the presence of vocalisations in each recorded hour per site. A mixed model was used to account for repeat sampling at sites. Time of sunset varied from 19:37 h to 20:40 h in spring/summer and from 17:06 h to 17:30 h in autumn/winter. We therefore standardised time as the hour since sunset with the hour that included sunset coded as ‘0’ and subsequent hours coded from 1 to 11. A cubic regression spline with shrinkage was applied to hour since sunset to account for non-linear patterns. Models were validated through visual inspection of residuals plotted against the fitted values and against each parameter within the model. Akaike Information Criterion backward selection was used to determine the most parsimonious model; where the least influential variable is dropped sequentially until the model no longer improves [44]. All models having a delta AIC <2 were considered to have support.

Prior to occupancy modelling, we checked for correlations between variables collected during each sampling period. Maximum nightly temperature was highly correlated with minimum nightly temperature (r_p_ = 0.92) and therefore was not included in our models. All other variables had correlation coefficients <0.6. All variables were scaled for inclusion within models. We used dynamic occupancy models and nightly presence/absence of vocalisations per site to determine the influence of season, lunar illumination (proxy for moonlight), nightly minimum temperature, nightly rainfall, inclement weather score and the interactions of season with each of rainfall, nightly minimum temperature, and inclement weather score, on the detection probability of Yellow-bellied Glider vocalisations (Table 1). Dynamic occupancy models were preferred over single season models due to the time interval (up to four months) between repeated sampling of the same site. Site occupancy, colonisation probability and extinction probability were held constant in all models. AIC backward selection was used as for our GAMMs to determine the most parsimonious model. We validated our models using the Mackenzie-Bailey fit statistic [45]. Model validation suggested that our data was slightly over-dispersed (*χ*^2^ = 60583.46, p = 0.081, ĉ = 0.168). We therefore included an over-dispersion parameter in our models and used Quasi Akaike Information Criterion corrected for small sample sizes [46]. To inform appropriate survey periods for Yellow-bellied Gliders we used our best supported models to predict the cumulative number of survey nights required to be 95% confident of a site-specific absence.

**Table 1:** The nightly mean, minimum and maximum values for inclement weather score, and climatic variables for the spring/summer and autumn/winter sampling periods, that were included in the dynamic occupancy models for Yellow-bellied Glider vocalisations.

Season	Parameter	Inclement Weather Score	Rainfall (mm)	Minimum nightly temperature (°C)	Lunar illumination (%)
Spring/summer	Mean	1.5	3.9	10.1	0.5
	Minimum	0.1	0	3.0	0
	Maximum	3	26.3	16.4	1
Autumn/winter	Mean	1.4	2.1	5.7	0.5
	Minimum	0	0	2	0
	Maximum	3	12.2	9.5	1

We further considered the influence of reducing the number of sampling hours per night to the first 3 or 4 hours from sunset (period of high calling activity of Yellow-bellied Gliders; [32]) on nightly detection probability.

Cumulative detection probability for a sampling period was calculated as:

$$Cumulative\;detection\;probability=1-\left(1-p_{1}\right)\times \left(1-p_{2}\right)\ldots (1-\;p_{n})$$

where *p*_1_ is the nightly detection probability and *n* is the total number of survey nights.

All analyses were conducted in R [47] with GAMMs run in ’gamm4’ [48], dynamic occupancy models in ’unmarked’ [49] and model selection and validation performed with ’AICcmodavg’ [50].

### Ethics statement

This study was conducted with the approval of the Deakin University Animal Ethics Committee (B24-2018) and under permits issued by the Department of Environment, Land, Water and Planning (*Wildlife Act* permit 10008841; *Forests Act* permit HUME-2018-01).

## Results

Yellow-bellied Glider vocalisations were present in 5.3% (284 of 5390) of survey hours. Naïve occupancy was 22 (51%) sites across both seasons; with 8 (19%) sites in both seasons, 19 (44.2%) sites in spring/summer and 11 (25.6%) sites in autumn/winter. At sites where gliders were detected, vocalisations were recorded during a mean 3.7 ± 0.7 SE (range 1 to 9) of survey nights in spring/summer, and 5.6 ± 1.00 SE (range 1 to 10) of survey nights in autumn/winter.

### Nightly and seasonal pattern of vocalisations

**Fig 3:**
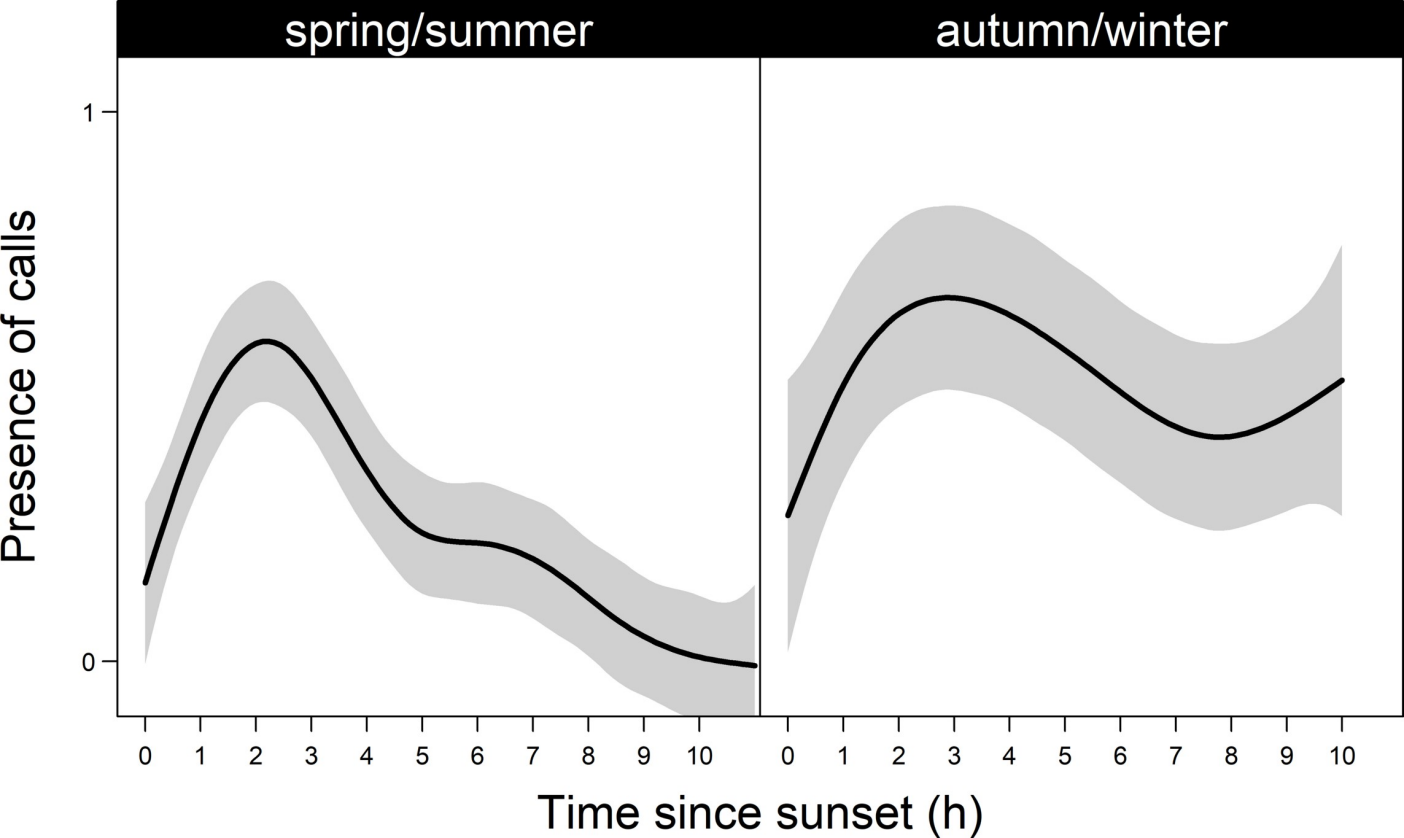
The number of sites (with 95% confidence intervals in grey) with Yellow-bellied Glider (*Petaurus australis*) vocalisations present per hour throughout the night in spring/summer and autumn/winter.

The presence of vocalisations in a recording was influenced by hour since sunset and the interaction of hour since sunset with season (AIC ω = 0.92; S1 Table). In both seasons, the number of sites with vocalisations present increased to reach a peak two to three hours after sunset. In spring/summer, the number of sites with vocalisations declined after this peak, whereas in autumn/winter, there was no discernible change (Fig 3). Count-based metrics suggest that the trend in vocalisations was similar between seasons (S1 Fig); however, this may have been biased by sites with numerous vocalisations. We were unable to account for this by using proportional metrics due to limited sample size. This model explained 18.1% of the variation in these data.

### Detection probability

**Fig 4:**
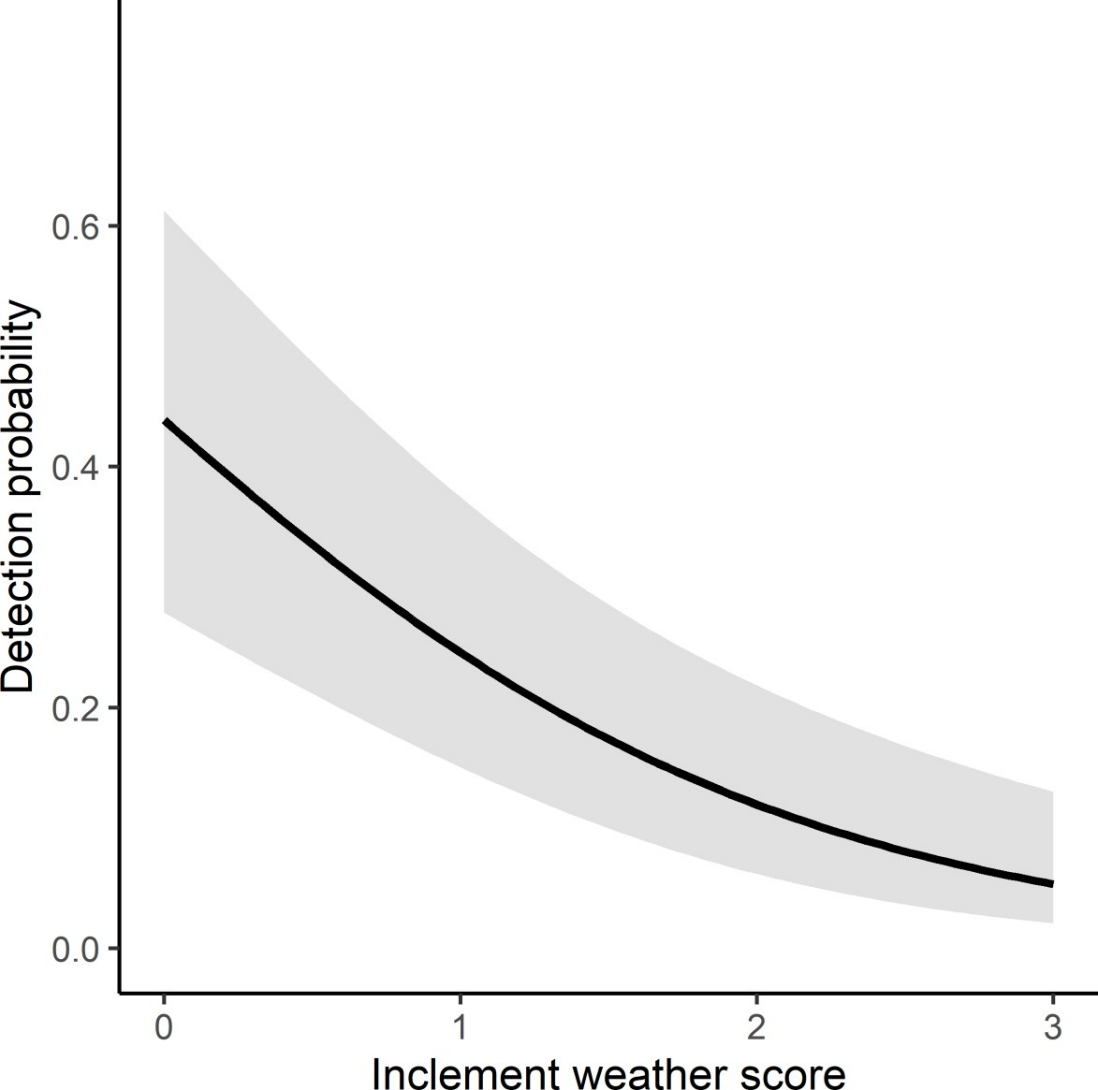
Influence of the inclement weather score on detection probability of Yellow-bellied Gliders (*Petaurus australis*). The score ranged from 0 (inclement weather score = 0) to 3 (inclement weather score = 3). 95% confidence intervals are shaded grey.

**Fig 5:**
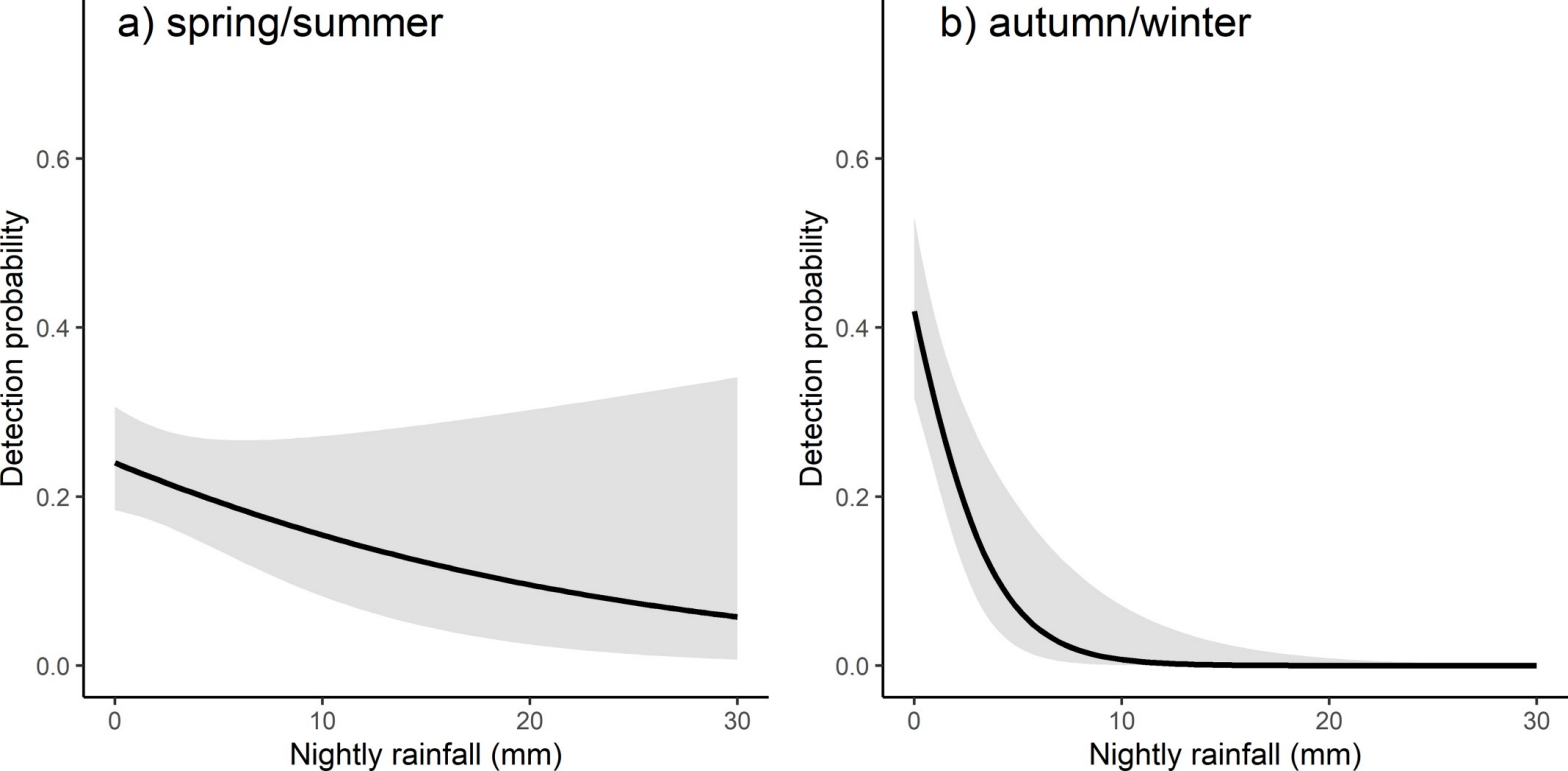
The influence of rainfall on detection probability of Yellow-bellied Gliders (*Petaurus australis*) during (a) spring/summer and (b) autumn/winter. 95% confidence intervals are shaded grey.

Our best supported model included the inclement weather score, and the interaction of season with rainfall (S2 Table). The inclement weather score had a negative influence on detection probability (β = -0.678, 95% CI: -0.392 to -0.964). Nightly detection probability declined from approximately 60% when the inclement weather score was zero to almost zero when there was a high inclement weather score (score = 3; Fig 4). There was no support for an influence of minimum nightly temperature (Δ QAIC = 2.89) or lunar illumination (Δ QAIC = 20.46, S2 Table) on detection probability. The interaction of season with rainfall had an influence on detection probability (β = 1.933, 95% CI: 0.706 to 3.160); rainfall reduced detection probability in autumn/winter more than in spring/summer (Fig 5 and Table 2). When considered as a main effect, increasing rainfall reduced detection probability (β = -2.193, 95% CI: -3.37 to -1.02). Season alone had no discernible influence on detection probability (β = 0.184, 95% CI: -0.51 to 0.87).

**Table 2:** The number of survey nights required to be 95% confident of a site-specific absence of Yellow-bellied Gliders, under varying conditions of season, inclement weather score and rainfall.

Variable	Value	Nightly detection probability (p)	Nights required to be 95% confident of a site-specific absence		
			Mean	Minimum	Maximum
Mean nightly inclement weather score	0	0.48	5	3.2	6.9
	1	0.28	10	7.0	12.2
	2	0.14	21	13.4	31.0
	3	0.06	47	22.6	98.8
Rainfall: spring/summer	Minimum (0 mm)	0.24	11	8.2	14.7
	Mean (3.9 mm)	0.22	13	9.2	16.5
	Maximum (26.3 mm)	0.06	51	7.2	416.1
Rainfall: autumn/winter	Minimum (0 mm)	0.42	6	4.0	7.9
	Mean (2.1 mm)	0.19	15	8.2	25
	Maximum (12.2 mm)	<0.001	>1000	-	-

### Influence of inclement weather and rainfall on surveys effectiveness

**Fig 6:**
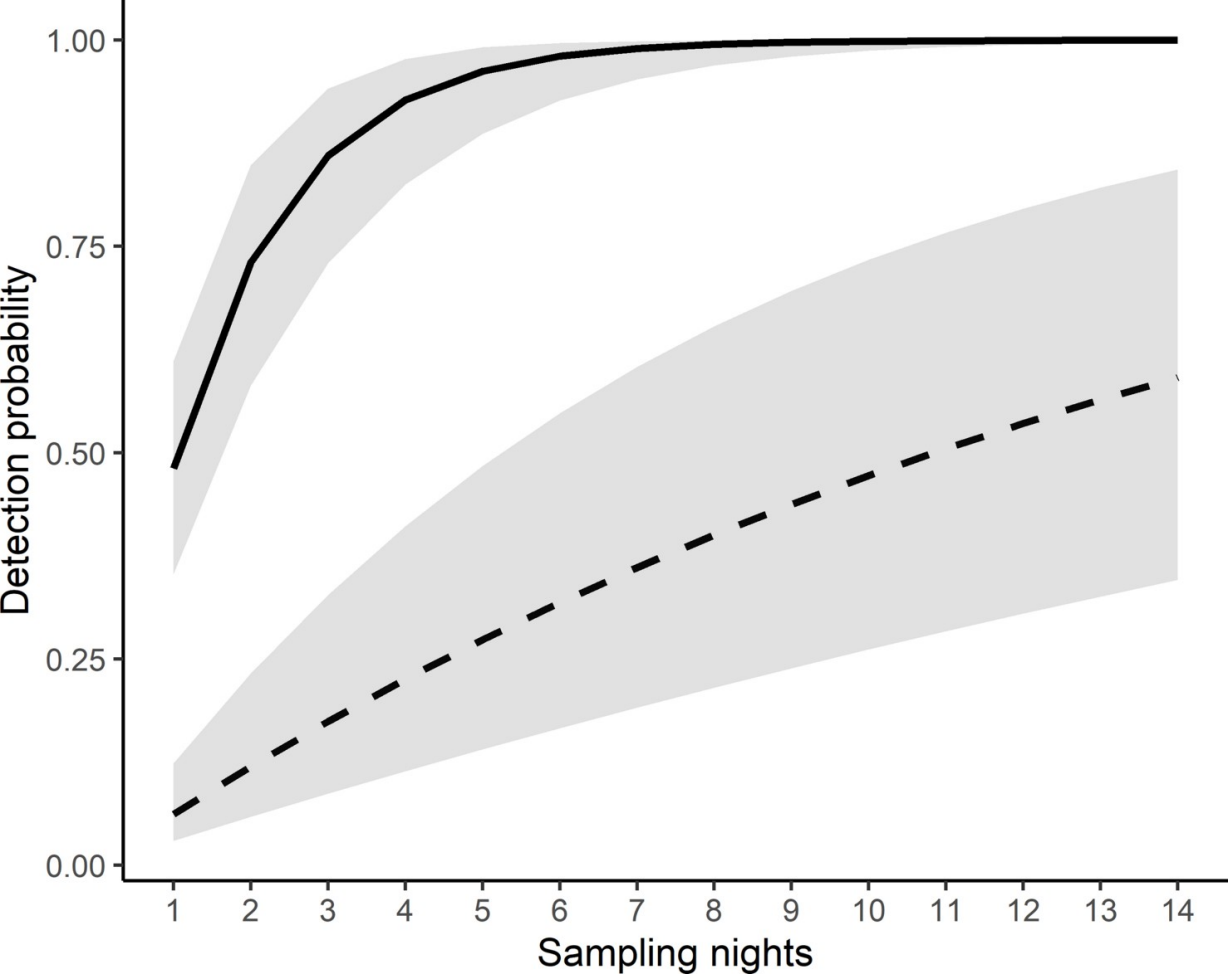
The relationship between the probability of detecting Yellow-bellied Glider vocalisations and the number of sampling nights with no inclement weather (mean score of 0; solid line) and frequent inclement weather (mean score of 3; dashed line). 95% confidence intervals are shaded grey.

When the mean nightly score for inclement weather was zero, five survey nights were required to be 95% confident of a site-specific absence (Fig 6 and Table 2). The number of survey nights doubled when the mean inclement weather score increased from 0 to 1, and then increased to ≥ 47 nights when the mean inclement weather score was 3. Rainfall also increased the number of sampling nights required to 13 nights in spring/summer, and to 15 nights in autumn/winter (Table 2).

### Optimising sampling efficiency

Recording for the first four hours (including the hour in which sunset occurred) per night had only a slightly lower nightly detection probability than sampling for 12 h per night, but the greatest cost-effectiveness for processing of recordings in both seasons (Table 3). When recording for four hours per night, an estimated total of 48 h of recording is required in spring/summer (i.e., 12 nights with four hours per night), and 24 h is required in autumn/winter (i.e., six nights with four hours per night) to be 95% confident of a site-specific absence of Yellow-bellied Gliders.

**Table 3:** Nightly detection probability, with the number of sample nights and total hours of recording required to be 95% confident of a site-specific absence of Yellow-bellied Gliders associated with recording for 3 h, 4 h and 12 h per night in spring/summer and autumn/winter.

Season	Hours recorded per night (h)	Nightly detection probability	Nights required to be 95% confident of a site-specific absence			Number of sample hours required
			Mean	Minimum	Maximum	
Spring/summer	3	0.10	27.2	15.7	48.1	84
	4	0.22	12	8.8	16.6	48
	12	0.23	9.6	7.6	12.3	120
Autumn/winter	3	0.32	7.8	5.6	10.9	24
	4	0.42	5.5	4.2	7.4	24
	12	0.40	5.8	4.6	7.5	72

Estimates assume that recording starts at the beginning of the hour that includes sunset.

## Discussion

We demonstrate that Passive Acoustic Monitoring (PAM) is an effective survey method for detecting the presence of Yellow-bellied Gliders, a highly vocal species that commonly occurs at low population densities [29–31]. PAM allows for multiple sites to be surveyed at the same time, and only requires one person to deploy and retrieve the ARUs, thereby being more cost-effective than traditional methods that rely on site visits over multiple nights. We found that Yellow-bellied Glider calls are easily detected in spectrograms of recordings when there is minimal ambient noise caused by rain and wind. Although manually searching spectrograms of recordings for vocalisations is an additional survey cost, it only takes a trained observer around one minute to process a one-hour recording. Automating call detection by developing an automated detection and classification algorithm would reduce the processing time but may be challenging for this species because of the wide variation in its calls [51].

Site occupancy in our study (26% in autumn/winter, and 44% in spring/summer) was similar to that recorded previously for Mountain Ash forest in the Central Highlands (33% occupancy reported in [52]). Absence of Yellow-bellied Gliders from many sites may be due to the area’s long history of timber harvest that has fragmented and reduced the total area and patch size of old-growth forest. Hollow-bearing trees and foods preferred by Yellow-bellied Gliders may be more abundant in old-growth forest than younger stands [52]. Large patches also are important in aiding dispersal [52]. Research to better understand the factors influencing site occupancy by Yellow-bellied Gliders in our study region is a topic for further research, and may be facilitated through the application of PAM, as developed in this study.

In both sampling seasons, we found that Yellow-bellied Glider calls were most common in the first four hours after sunset [28, 32, 53]. Yellow-bellied Gliders call as they emerge from their dens [32], and when gliding between trees. A second peak in calling also has been observed at sunrise when gliders are returning to their dens [32]. We recorded until after sunrise in the spring/summer sampling period but for unknown reasons, did not observe a second peak. We therefore recommend only recording during the first four hours after sunset, which both saves power and digital storage, and results in fewer recordings that must be processed.

Although Yellow-bellied Gliders are known to vocalise year-round, we observed higher abundance of vocalisations in autumn/winter than in spring/summer. This may have been due to seasonal variation in gliding behaviour due to the distribution and quality of food resources [28, 34, 35, 53]. Yellow-bellied Gliders prefer to feed on sap and nectar, but they supplement their diets with arthropods which provide an important source of protein [28]. Because resources such as sap and nectar tend to be clumped, Yellow-bellied Gliders spend less time gliding (and therefore calling) when feeding on this resource [4, 34]. We did not assess food resource availability in our study area, but it is possible that season influenced the type and distribution of food resources used (e.g. [53, 54]) and hence calling behaviour.

Of the other variables we tested (i.e., rainfall, minimum nightly temperature, lunar illumination), only rainfall had an influence on calling activity, negatively influencing detection probability. Rainfall previously has been identified as a factor influencing detection probability [4], and surveying when it is raining is usually avoided [37, 38]. In our study, the negative influence of rainfall was more pronounced during autumn/winter than spring/summer. This may have been a thermoregulatory response to the combination of rain and lower temperatures. Yellow-bellied Gliders may have a limited energy budget due to their diet; it is therefore not surprising that its activity (and hence calling activity and detection probability) is lower under wet and cold weather conditions when the energetic cost of thermoregulation may be higher. We did not record any influence of temperature on calling activity; however, our temperature data was obtained from nearby weather stations which may not accurately reflect local temperatures. Similarly, our estimate of lunar illumination may not have accurately represented the amount of moonlight at each site, which may have influenced our results. Future studies should consider recording these variables at the site level.

Inclement weather resulting in high levels of ambient noise is a factor that must be considered in the use of PAM for survey of any terrestrial species (e.g. [23]). Ambient noise limits an observer’s ability to see calls in spectrograms, and the effectiveness of automated call recognition [55]. In our study, inclement weather score had a considerable influence on Yellow-bellied Glider detection probability. For PAM to be effective as a survey method for Yellow-bellied Gliders, conducting surveys during periods of inclement weather should be avoided.

At many of the sites where we detected Yellow-bellied Gliders, calling activity was low with vocalisations detected in fewer than half of the survey nights, and sometimes in just a few one-hour recordings. Despite this, we found that an ARU programmed to record continuously for four hours from sunset and deployed at a site for six nights in autumn/winter and 12 nights in spring/summer provides 95% confidence of a site-specific absence of Yellow-bellied Gliders.

## Conclusions

Despite concerns that populations of Yellow-bellied Gliders are declining, there remains a paucity of knowledge on the population dynamics and status of individual populations. Implementation of PAM for Yellow-bellied Gliders will allow for more efficient and robust assessment of habitat use and the influence of threatening processes on populations. For PAM to be effective for determining the presence or absence of Yellow-bellied Gliders in a site and at any time of year, we recommend deploying ARUs for six nights of good weather conditions (calm or light wind, and no rain). If the weather forecast includes some nights of windy or wet conditions, this survey period should be increased accordingly. Extended periods of poor weather conditions should be avoided. ARUs should be programmed to record for four hours beginning from the hour of sunset. Based on Yellow-bellied Glider calls being audible to approximately 400 m, each ARU will have a detection area of around 50 hectares. Further research is necessary to determine if PAM can be modified to estimate abundance of Yellow-bellied Gliders, either by determining the relationship between calling rate and abundance (e.g. elephants, [56]; some bird species, [57], or using arrays of ARUs to determine the location of calls for distance-based abundance estimates [58].

## Supporting information

S1 Fig The mean number of sampled hours per site (with 95% CI) in which Yellow-bellied Glider vocalisations were present. (TIF)

S1 Table Model selection table for Yellow-bellied Glider generalised additive mixed models. Variables included are presence or absence of yellow-bellied glider calls per hour (Calls), time since sunset (TSS), and the survey season (season). Reported are the number of parameters (K), Akaike Information Criterion, corrected (AICc), delta AIC, AIC weightings and loglikelihood (LL).
(DOCX)

S2 Table Model selection table for Yellow-bellied Glider dynamic occupancy models. Model parameters include site occupancy (psi), site colonisation probability (g), site extinction probability (e), and detection probability (p). Reported are the number of parameters (K), Quasi Akaike Information Criterion corrected (QAICc), delta QAIC, QAIC weights and QAIC loglikelihood. Variables include the survey season (season), the nightly rainfall (rain), the minimum nightly temperature (mintemp), the proportion of lunar illumination (illum) and inclement weather score (IW).
(DOCX)

S1 File Presence/absence of Yellow-bellied Glider calls and covariates per night at each site in spring/summer and autumn/winter. A description of each worksheet is given in the first worksheet ‘Codes’.
(XLSX)
