# Supplementary figures and images for: Passive acoustic monitoring for detecting the Yellow-bellied Glider, a highly vocal arboreal marsupial
Source: PLoS One. 2021 May 25;16(5):e0252092. doi: 10.1371/journal.pone.0252092 (PMC8148312; doi:10.1371/journal.pone.0252092)

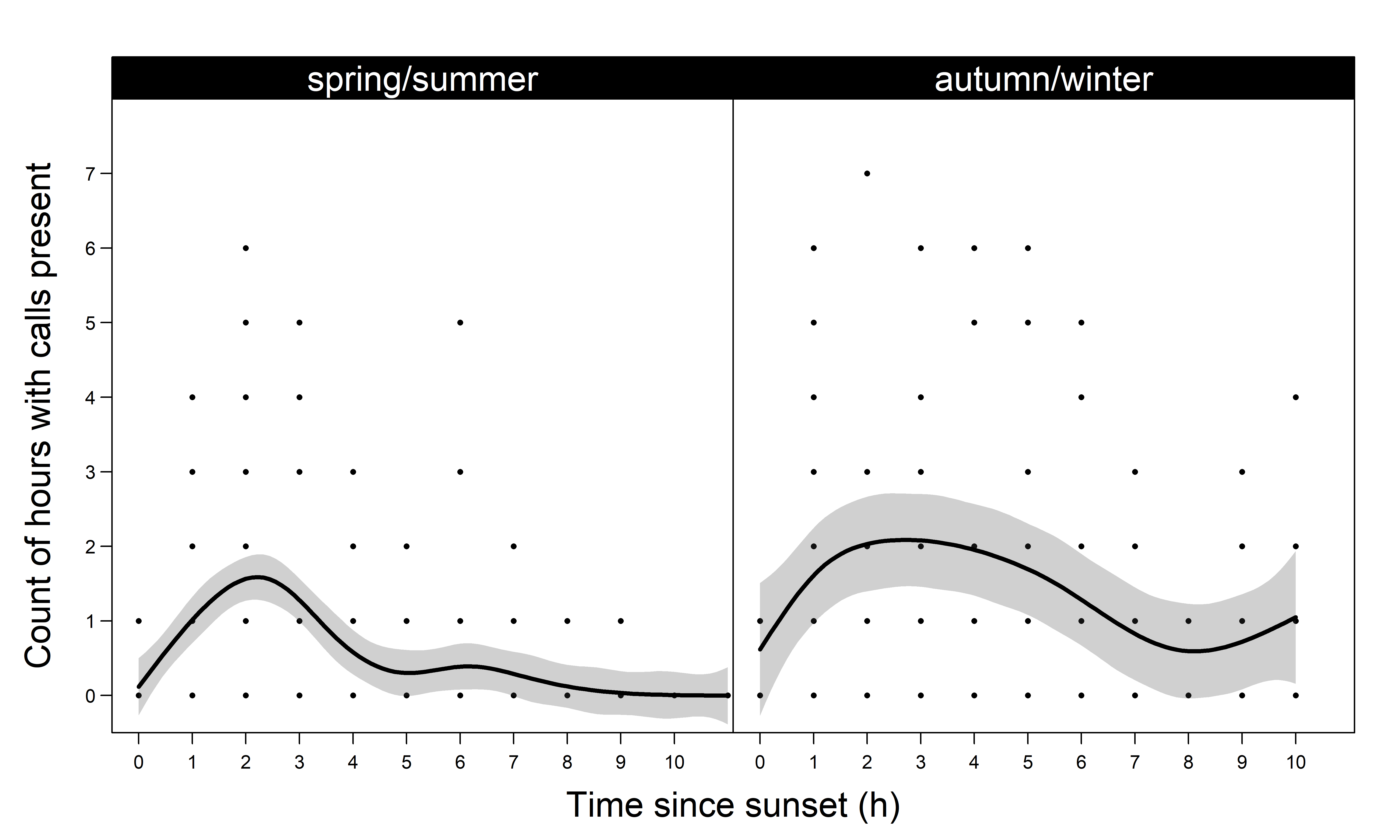

Supplement: S1 Fig — (TIF) [file pone.0252092.s001.tif]
