# Supplementary material for: Passive acoustic monitoring for detecting the Yellow-bellied Glider, a highly vocal arboreal marsupial
Source: PLoS One. 2021 May 25;16(5):e0252092. doi: 10.1371/journal.pone.0252092 (PMC8148312; doi:10.1371/journal.pone.0252092)
Supplement: S1 Table — Variables included are presence or absence of yellow-bellied glider calls per hour (Calls), time since sunset (TSS), and the survey season (season). Reported are the number of parameters (K), Akaike Information Criterion, corrected (AICc), delta AIC, AIC weightings and loglikelihood (LL). (DOCX) [file pone.0252092.s002.docx]

| Model | K | AICc | ∆ AIC | AIC ω | LL |
| --- | --- | --- | --- | --- | --- |
| Calls ~ s(TSS*season) + season | 5 | 385.69 | 0.00 | 0.92 | -187.77 |
| Calls ~ (TSS) + season | 4 | 390.58 | 4.89 | 0.08 | -191.24 |
| Calls ~ s(TSS) | 3 | 417.99 | 32.30 | 0.00 | -205.96 |
| Calls ~ season | 3 | 436.58 | 50.89 | 0.00 | -215.26 |

**S1 Table. Model selection table for Yellow-bellied Glider Generalised Additive Mixed Models.** Variables included are presence or absence of yellow-bellied glider calls per hour (Calls), time since sunset (TSS), and the survey season (season). Reported are the number of parameters (K), Akaike Information Criterion, corrected (AICc), delta AIC, AIC weightings and loglikelihood (LL).
