# Supplementary material for: Passive acoustic monitoring for detecting the Yellow-bellied Glider, a highly vocal arboreal marsupial
Source: PLoS One. 2021 May 25;16(5):e0252092. doi: 10.1371/journal.pone.0252092 (PMC8148312; doi:10.1371/journal.pone.0252092)
Supplement: S2 Table — Model parameters include site occupancy (psi), site colonisation probability (g), site extinction probability (e), and detection probability (p). Reported are the number of parameters (K), Quasi Akaike Information Criterion corrected (QAICc), delta QAIC, QAIC weights and QAIC loglikelihood. Variables include the survey season (season), the nightly rainfall (rain), the minimum nightly temperature (mintemp), the proportion of lunar illumination (illum) and inclement weather score (IW). (DOCX) [file pone.0252092.s003.docx]

| Model | K | QAICc | ∆ QAIC | QAIC ω | QLL |
| --- | --- | --- | --- | --- | --- |
| psi(.)g(.)e(.)p(season*rain + rain + IW) | 9 | 353.35 | 0.00 | 0.69 | -164.95 |
| psi(.)g(.)e(.)p(season*rain + rain + IW + mintemp) | 10 | 356.24 | 2.89 | 0.16 | -164.68 |
| psi(.)g(.)e(.)p(season + rain + IW) | 8 | 357.00 | 3.64 | 0.11 | -168.38 |
| psi(.)g(.)e(.)p(season*rain + rain + IW + season*mintemp + mintemp) | 11 | 359.41 | 6.06 | 0.03 | -164.45 |
| psi(.)g(.)e(.)p(season*rain + rain + IW + season*mintemp + season*IW + mintemp) | 12 | 363.15 | 9.80 | 0.01 | -164.38 |
| psi(.)g(.)e(.)p(season*rain + season*mintemp + season*IW + rain + IW + Illum + mintemp) | 13 | 373.81 | 20.46 | 0.00 | -167.63 |
| psi(.)g(.)e(.)p(.) | 5 | 384.91 | 31.55 | 0.00 | -186.64 |

**S2 Table. Model selection table for Yellow-bellied Glider dynamic occupancy models.** Model parameters include site occupancy (psi), site colonisation probability (g), site extinction probability (e), and detection probability (p). Reported are the number of parameters (K), Quasi Akaike Information Criterion corrected (QAICc), delta QAIC, QAIC weights and QAIC loglikelihood. Variables include the survey season (season), the nightly rainfall (rain), the minimum nightly temperature (mintemp), the proportion of lunar illumination (illum) and inclement weather score (IW).
